# Imagining emotions in storyworlds: physiological narrated perception and emotional mental imagery

**DOI:** 10.3389/fnhum.2024.1336286

**Published:** 2024-01-24

**Authors:** María-Angeles Martínez

**Affiliations:** Department of Modern Philology, Universidad de Alcalá, Alcalá de Henares, Spain

**Keywords:** emotional mental imagery, emotion schemata, physiological narrated perception, embodiment, narrative experiencing

## Abstract

Research into narrative experiencing acknowledges the role played by mental imagery in readers’ emotional responses and feelings of embodiment. In narratives, mental imagery is frequently evoked through narrated perception, or the textual presentation of sensory perception, as in “The silence in the house was complete”. Narrated perception spans the five senses – sight, hearing, smell, touch, and taste; however, little attention has been paid to the narrated description of characters’ basic physiological processes, that is, those connected to the invisible systems – respiratory, digestive, cardio-vascular, muscular – and their relevance to the mental construction of narrative emotions. This study explores the presentation of fictional characters’ physiological processes as a prompt for readers’ embodied experience of storyworlds through the metonymic activation of self-relevant emotion schemata. To this purpose, the presentation of characters’ internal physiological processes in two fictional samples of similar length – Rosemary Timperley’s 1955 ghost story “Harry” and chapter one in Ewan McEwan’s 2002 novel *Atonement* – is analysed. The findings suggests that these descriptions enrich the imagined nature of narrative emotions through underspecification, and increase opportunities for perceived self-relevance and engagement.

## 1 Introduction

There is widespread academic agreement on “the emotional nature of mental imagery” and its power to evoke emotion (Blackwell, 2020: 241). Accordingly, research into embodied narrative experiencing highlights the importance of the textual mechanisms whereby mental imagery can be prompted in readers (Kuzmičová, 2014; Otis, 2022). Paramount among these is *narrated perception*, or the textual presentation of “sensory perceptions within what looks like narration” (Pallarés-García, 2012: 170). Consider, for instance, the visual and olfactory mental imagery invited by “Light filtered through the essence of lemons” (Durrell, 1968[1957]: 18). Five types of narrated perception are usually acknowledged (Fludernik, 1996: 305−309; Shen, 2005; Pallarés-García, 2012; Martínez, 2018: 73−75), corresponding to the five senses, namely, visual, olfactory, auditive, tactile, and gustatory. The narrated representation of these sensorial perceptions can be expected to have profound effects on readers’ emotional responses, both mimetic with the perceiving narrative perspectivizer – focalizer – or, simply, felt for themselves (Miall and Kuiken, 2002). However, little attention has been paid to the role of the description of inner body perceptions, such as “Then I heard *something that chilled my blood*” (my italics) (Timperley, 1983[1955]: 46), in the generation of mental imagery and imagined emotions. The present study investigates the presence of this kind of physiological description, which I call *physiological narrated perception*, in narrative language, and its role in the metonymic activation of emotion schemata (Kövecses, 2014), or complex mental models containing background information related to the causes, symptoms, and effects of emotions.

The analysis focuses on the cases of physiological narrated perception, such as the above, found in two narrative samples of similar length from two very different genres: the ghost story “Harry” (Timperley, 1983[1955]) and the first chapter in Ewan McEwan’s novel *Atonement* (2002). The aim is to compare the presence of physiological description in these two narrative samples, considering its potential connection to narrative emotions and narrative meaning construction. In this respect, the ghost story is expected to contain a larger number of these physiological descriptions, given that fear is considered a basic emotion (Gu et al., 2019) whose symptoms (Stemmler, 2004) might be easily recognized by readers. *Atonement*, on the contrary, involves a stronger psychological complexity which physiological narrated perception can only ambiguously represent and help to imagine, so that the characters’ emotions can be expected to be more explicitly identified. Since psychological and neuroscientific evidence suggests that “the distinction between remembering and imagining is far more blurred than is typically assumed” (Abraham, 2020: 5), narrated physiological perception may play a crucial role in the activation of emotion schemata based on remembered – personally lived, observed, reported – emotional experiences. The findings seem to confirm this hypothesis, and to further suggest that the narrated description of the focalizing characters’ physiological processes and internal sensory experience tends to co-occur with the narrative presentation of different types of external sensorial experience. This suggests that the concept of narrated perception should be enlarged to encompass not exclusively the external senses, but also the workings of the body’s invisible systems, since attention to physiological narrated perception can enhance the narratological, stylistic, psychological, and neuroscientific study of embodied narrative experiencing, and of the imagined and felt emotions which characterize it.

The study begins with an overview of the concept of mental imagery at large, and emotional mental imagery in particular. Then, the embodied nature of narrative experiencing and the use of narrated perception as a prompt for readers’ generation of self-relevant emotional mental imagery is briefly presented. The role of the narrated description of emotion-related physiological activity is subsequently discussed with reference to the concept of emotion schemata. This is followed by a brief account of the methodology used in the analysis, a discussion of its results and findings, and some concluding remarks.

## 2 Emotional mental imagery

Conceptualizing human imagination is not an easy task. As Abraham (2020: 1) notes:

“The conceptual space it [imagination] spans is stupendously vast, stretching across the real and the unreal, the possible and the impossible. Its workings are spontaneous and deliberate, ordinary and extraordinary, conscious and unconscious, deriving from the outer world external to our bodies as well as our inner world.” (Abraham, 2020: 1).

As can be observed, the conceptual boundaries of human imagination are far from clearly defined, and neuroscience highlights its frequent overlaps with, for instance, memories (Schacter and Addis, 2020) and beliefs (Meskin and Weinberg, 2011). The generation of mental imagery (Pearson and Kosslyn, 2013; Kuzmičová, 2014; Otis, 2022), however, is one of the cognitive activities in which imagination, understood as the “ability to conjure up images, ideas, impressions, intentions and the like” (Abraham, 2020: 1), plays an outstanding role. According to Pearson et al. (2015: 590), the term ‘mental imagery’ can be used “to refer to representations and the accompanying experience of sensory information without a direct external stimulus.” These mental images are “recalled from memory and lead one to re-experience a version of the original stimulus or some novel combination of stimuli” (Pearson et al., 2015: 590). In other words, the generation of mental imagery may involve the re-experiencing of sensory representations retrieved from memory, as well as their re-combination “with other representations and knowledge to produce an image of a scene or object that has never been experienced” (Blackwell, 2020: 242). This need not happen voluntarily, as “external events or internal associations also can trigger a mental image, even if one does not want to experience the image at that time” (Pearson et al., 2015: 590). Furthermore, “imagining this scene and experiencing the sights, sounds, and other sensations in imagination will involve very similar patterns of neural activation as if the scene was being perceived and experienced in reality” (Blackwell, 2020: 242). As Blackwell (2020: 243) explains, “mental imagery of emotional events often contains representations of emotional states, resulting in an experiencing these states,” and is strongly subjected to individual variation.

For instance, Blackwell (2020: 242) uses the examples of mentally rehearsing an upcoming job interview by picturing the room, sounds, or faces to be encountered, or involuntarily evoking a pleasant moment when hearing a certain song on the radio. Emotional mental imagery can also involve distressing negative imagery, related to traumatic past events or to anxious anticipation of future ones, and has been used therapeutically to reduce fear responses through imaginal exposure, or to reduce the emotional impact of a traumatic experience (Blackwell, 2020: 245−247). Given the strong power of mental imagery to evoke emotion (Stemmler, 2004; Holmes and Matthews, 2005; Stemmler et al., 2007; Suess and Rahman, 2015; Blackwell, 2020: 242), this re-experiencing can be expected to include the emotional states with which the real-world sensorial perception is associated, commonly multimodally (Kuzmičová, 2012; Lacey and Lawson, 2013; Otis, 2022), to be either re-lived in their original form, or re-combined in novel forms driven by contextual relevance.

## 3 Narrated perception and the narrative prompting for mental imagery

### 3.1 Narrative experiencing and embodiment

The relevance of mental imagery to the study of narrative emotions is undeniable, due to its bearing on the embodied nature of narrative experiencing. As Johnson (2007: 10) remarks, an understanding of human cognition as an embodied phenomenon “situates meaning within a flow of experience that cannot exist without a biological organism engaging its environment.” The belief that human cognition is embodied, in the sense that higher order thought cannot be disconnected from brain and body (Johnson, 1990, 2007, 2018), nor from the environmental features that surround an organism (Barsalou, 2010) has become a central tenet not only in neuroscience (Gallese and Goldman, 1998; Gallese and Snigaglia, 2011; Gallese, 2017), but also in philosophy, linguistics, and narrative theory. In fact, cognitive narratology draws on embodied cognition in its view of the storyworld (Herman, 2002) as the situational model of an imaginary state of affairs which functions as a mentally constructed simulation environment for the training and learning of personally relevant behaviors and emotions (Mar and Oatley, 2008). A situational model (Johnson-Laird, 1983) is a complex set of interrelated schemata which, in narrative experiencing, intervenes in the interactants’ co-conceptualization of “how referents stand in particular relation to one another in the narrated world, (and) how some of these referents can be construed as participants more or less centrally involved in states of affairs, processes, events” (Herman, 2002: 20). Within this view, narrative engagement is an embodied cognitive experience which requires audience members – readers, cinema goers, video-game players – to deictically shift (Duchan et al., 1995) into the person, time, and place parameters which define the deictic center occupied by the narrative entity that provides perspective on the storyworld, be it a narrator or a focalizing character, in order to co-experience the fictional events and situations. This involves “mentalizing strategies, such as imaginatively adopting the stance of another person” (Lamm et al., 2019: 50), including their emotional states and evaluative positioning.

Neuroimaging methods have provided evidence that the brain areas connected to certain actions and bodily phenomena are also neurally activated upon observation of those actions and bodily phenomena in others (Gallese and Goldman, 1998). Although, as Lamm et al. (2016) observe (2016: 1), “the relationship between neural activations and mental representations (…) is not always straightforward,” so that (cognitive) mental representations cannot be solidly inferred from shared neural activation, a vast body of related research, particularly within Theory of Mind and theories of embodied cognition, has been built around mirror neurons and their implications for the mentalizing of other people’s mental states from their observed bodily behavior. It is here that the significance of the narrative representation of external sensory experience – visual, auditive, olfactory, tactile, gustatory – becomes apparent. Equally important, though, is the narrative presentation of internal somatosensory experience, connected to the invisible systems – cardiovascular, digestive, respiratory, muscular, homoestatic – and to bodily sensations such as pain or hunger, given their crucial association with, and indexing of, emotional experience (Stemmler, 2004; Stemmler et al., 2007; Suess and Rahman, 2015).

### 3.2 Narrated perception

In her discussion of the role of mental imagery in literary narratives, Kuzmičová (2014: 275) refers to “those instances in which modern silent readers of literary narrative, while reading an expression “X,” experience some form of sensory representation of what they (more or less literally) understand to be X.” Although Kuzmičová’s account pays due attention to both external and internal sensorial experience, the stylistic study of the narrated representation of this experience revolves around the concepts of “narrated perception” (Cohn, 1978: 111; Fludernik, 1996: 305−309; Shen, 2005; Pallarés-García, 2012; Martínez, 2018: 73−75), or represented perception” (Brinton, 1980; Van Krieken, 2018), which traditionally encompasses the narrative presentation of experience perceived through the external senses.

Narrated perception is defined as “sensory perceptions within what looks as narration” (Pallarés-García, 2012: 170), and is one of the narrative techniques used in the rendering of consciousness (Cohn, 1978: 111). Instances of narrated perception portray “the sensory perceptions of a fictional character by describing events as they are experienced by that character” (Fludernik, 1996: 305−309), and are not introduced by a verb of cognition such as “saw” or “heard,” so that “objects, people, events and phenomena (for example, sounds, smells) are described just as they would have looked or felt to the character concerned” (Pallarés-García, 2014: 19). In her empirical study on perception attribution, Van Krieken (2018) investigated the extent to which the absence of viewpoint markers such as “saw” or “heard” influenced readers’ perceptual attributions, and found that “in the absence of a contextual viewpoint marker, they (readers) are more likely to attribute observations to the narrator (than to a character) and to refrain from clearly attributing observations to either the narrator or the character” (2018: 783),” resulting in increased ambiguity. In other words, the absence of a perceptual verb increases readers’ cognitive effort, because it involves the need to assess the likelihood that the described perception is attributed to the focalizing character. This higher cognitive demand can, however, have desirable effects on narrative engagement, since readers are forced to more vividly resort to mental imagery, as perceptual salience falls on the perceived rather than on the perceiver.

Narrated perception can be visual as in (1), olfactory as in (2), auditory as in (3), tactile as in (4), and gustatory as in (5). In all cases, the italics are mine unless otherwise stated:

(1) *A scatter of bright uneven grass splashed the bald brown patches of earth.* (Timperley, 1983[1955]: 45) (Visual).
(2) *Some small something chattered in a tree*. [Lindsay, 2004: 6; in Martínez (2018): 74] (Auditory).
(3) An air full of brick-dust – *sweet-smelling brick-dust and the odour of hot pavements slaked with water*. (Durrell, 1968[1957]: 18; in Martínez (2018): 74) (Olfactory).
(4) Mrs. Tallis read the seven pages of *The Trials of Arabella* (italics in the original) in her bedroom, at her dressing table, *with the author’s arm around her shoulder* the whole while. (McEwan, 2002: 4) (Tactile).
(5) *The sweetness* [of tea] did not conceal a taste of disinfectant. (McEwan, 2002: 224) (Gustatory).

Some of these narratorial presentations of a character’s sensorial experience, however, involve the inner-body perception of physiological, somatic processes, as in (6):

(6) *My hands were trembling*. (Timperley, 1983[1955]: 45).

These somatic processes are not traditionally considered cases of narrated perception, but they strongly encourage readers not only to neurally share the character’s experience of a bodily process, but also to infer the emotions and mental states which these bodily reactions metonymically represent. In example (6), from the ghost story “Harry,” the first person narrator, mother of an adopted six-year-old girl, Christine, has just discovered some crucial information which, in her view, supports her deep belief that her little daughter’s imaginary friend Harry is, actually, the girl’s dead brother’s ghost. The description of this somatomotor, muscular self-perception comes near the end of the narrative, when readers are immersed in a ghostly scenario which the mother firmly constructs as real (Martínez, 2022). Within this ghost frame, readers’ mentalizing of the character’s bodily sensation of muscular tremor is likely to include the inference of feelings of horror and shock as the reason for the self-reported muscular malfunction. Such reports of physiological activity must definitely have a strong bearing on readers’ emotional mental imagery and embodied experience of the storyworld, not just through empathy, or feeling what the character feels (Keen, 2010), but also, and most importantly, through the activation of relevant emotion schemata and the generation of fresh emotions (Miall and Kuiken, 2002), connected to what individual readers may imagine that they would be feeling in the environmental circumstances which surround the character within the ontology of the storyworld.

## 4 The metonymic triggering of emotion schemata

Emotion schemata are complex mental models which contain individuals’ background information related to the causes, symptoms, and effects of emotions (Kövecses, 2000; Bednarek, 2009), and which are built from personal embodied experiences within socio-cultural settings. As Bednarek notes (2009: 397), “These schemata are presumably based on people’s actual emotional experience (e.g., increased body heat when angry), and observing it in others (perception) as well as exposure to discourse on emotions and other socializing processes.” Although some of these emotion schemata are social (Kövecses, 2000), those connected to bodily experiences of fatigue, hunger, or physical pain, which Gallese (2010: 59) describes as “psychological states triggered by physiological states,” can be expected to be universal, and thus have the potential to evoke similar emotions in large numbers of readers. The four basic, or primitive emotions (Ekman, 1992), namely, fear, anger, joy, and sadness, are also considered universal, and even shared with other living organisms, due to their likely phylogenetic origins in survival mechanisms not exclusively human (Gu et al., 2019). Emotion schemata are frequently triggered by the use of emotion language (Bednarek, 2009), which explicitly refers to a given emotion, as in examples (7) and (8). Here, the elicited emotions are, respectively, FEAR and DESPAIR:

(7) (…) yet the first time Christine mentioned the name, I felt a premonition of *fear*. (Timperley, 1983[1955]: 33).
(8) Don’t be silly. Lots of children have an imaginary companion,’ I told myself *desperately.* (Timperley, 1983[1955]: 35).

However, given the quasi-universal nature of lower order cognition (Rietveld et al., 2018: 49), that is, cognition involving “anatomical structure, body parts, sensorimotor contingencies, and action capabilities,” as well as hormonal and neurotransmitter levels, or body chemistry (Kirchhoff, 2018: 363), it can be expected that the narrative representation of the perspectivizing character’s physiological processes, that is, those connected to the invisible systems – respiratory, digestive, muscular, cardio-vascular – will similarly evoke an emotional state, by metonymically pointing to the emotion by which those physiological processes are caused within relevant emotion schemata.

This metonymic pointing is triggered by the connections between a bodily reaction and an emotion. As noted by Kövecses (2014: 17), this is as an instantiation of the EFFECT OF THE EMOTION FOR THE EMOTION metonym, governed by the cognitive principle of relative salience for metonymic structure which Radden and Kövecses (2007: 20) identify as CONCRETE OVER ABSTRACT, entailing the connected VISIBLE FOR INVISIBLE. According to Radden and Kövecses (2007: 20), “(…) body parts make particularly “good” objects, and we routinely access various abstract human domains by reference to our body.” In this sense, readers’ mentalizing of the emotions that make a character claim *My hands were trembling*, as in example (6) above, in the context of a ghost story, is likely to cause the metonymic triggering of their emotion schemata for “fear”, one of the basic emotions (Ekman, 1992; Gu et al., 2019), to be fleshed out on the basis of the individual’s physiological, socio-cultural, and narrative experiences. These are some common conceptual metonymies for emotions:

BODY HEAT FOR ANGER (being a *hothead*)
DROP IN BODY TEMPERATURE FOR FEAR (getting *cold feet*)
CHEST OUT FOR PRIDE (*puffing one’s chest out* with pride)
RUNNING AWAY FOR FEAR (*fleeing* the scene)
WAYS OF LOOKING FOR LOVE (*looking* at someone *amorously*)
FACIAL EXPRESSIONS FOR SADNESS (*having* a sad *face*)
(Kövecses, 2014: 17)

The immersive power of these metonymic triggers lies in the chance that a given physiological reaction can be associated with different emotion schemata by different readers, thus increasing opportunities for personally relevant meaning construal operations. For instance, readers of example (6) – *My hands were trembling* – may activate their emotion schema for FEAR as the default emotion connected to this physiological perception, given that they are experiencing a ghost story. But, even in a ghostly context, the emotion can also be conceptualized as something more than, or something similar to fear. Moreover, even in readers in which this bodily perception imaginatively evokes ‘fear,’ the specifics of the FEAR emotion schema are likely to differ in many ways, not only because of the idiosyncratic nature of mental schemata (Bartlett, 1932; Rumelhart, 1980), but also because, as Blackwell (2020: 250) points out, “There appear to be many important individual differences relating to the generation or experience of mental imagery of specific emotional valence.”

## 5 Materials and methods

In order to gain a better understanding of the role of physiological narrated perception in the metonymic activation of emotion schemata and the generation of imagined emotions by readers during fictional narrative experiences, Rosemary Timperley’s short story “Harry” (1955) and the first chapter in Ian McEwan’s novel *Atonement* (2002) have been analysed. “Harry” is a 15-page long ghost story, originally published in Asquith (1955) *The Third Ghost Book*, and a regular in ghost story collections such as Dahl (1983) *Book of Ghost Stories*, and Penzler (2012) *The Big Book of Ghost Stories*. It tells the story of a little girl, Christine, whose adoptive mother suspects is being haunted by her deceased brother Harry. “Harry” has also been adapted to the television screen as a chapter in the Canadian series *First Person* (1960−1961), and made into a short film entitled *Twice Removed* (2003). *Atonement*, on the other hand, tells the story of the Tallis sisters, Briony and Cecilia, starting in 1934’s pre-World-War I Britain. Its first chapter is a 14-page long extract, similar in length to “Harry,” which makes it suitable for the comparative analysis proposed. In “Harry,” the narrative is focalized by the first person narrator Mrs. James, Christine’s mother, while in *Atonement* the narrator is omniscient, but often resorts to third-person focalization – that is, the use of a character’s consciousness as the vantage point on the storyworld into which readers have to deictically shift. In chapter one, this focalizer is eleven-year old Briony Tallis, with some narratorial interference.

The first step in the analysis has been to identify the cases of emotion language, or “lexical items that denote emotional experience for example *love*, *hate*, *joy*, *envy*, *sad*, *mad*, *enjoy*, *dislike*, etc., (as well as fixed expressions such as *He had a broken heart*)” (italics in the original) (Bednarek, 2009: 398) in the two narratives. Then, the study has focused on the presence of physiological narrated perception, or the narrated description of physiological processes associated with characters’ emotional states, in the two samples. Since “Harry,” the ghost story, usually appears in ghost story collections, its readers are likely to be aware that fear is a predictable genre-related emotion. This will probably help them predict this emotion in the main character and first person narrator, Mrs. James, who firmly believes that her adopted five-year-old daughter, Christine, is being haunted by the ghost of her deceased brother. In other words, in the ghost story, fear and fear-related emotions are strong candidates for metonymic activation through the narrated perception of the character’s somatosensory reactions, on the basis of the ghost story genre’s association with heightened sensorial perception, embodied experience, and emotional delusion (Li, 2021). *Atonement*, on the contrary, is a high-brow novel and an academic favourite due to the complexity of the emotional turmoil which triggers its sequence of tragic events. This complexity is further complicated by narratorial unreliability (Phelan, 2005), so that readers may find it difficult to infer the exact emotions felt by young Briony in her pre-adolescent awakening. Accordingly, the research hypothesis is that the ghost story will rely on the metonymic triggering of emotion schemata through physiological narrated perception more frequently than the novel chapter, in which emotions may need to be more clearly identified through the use of explicit emotion language.

## 6 Analysis and discussion

The analysis shows that, from a total 99 occurrences of expressions denoting emotional states, both explicit and metonymic, in the narrative texts analysed, there are 57 cases of physiological narrated perception (57,57%), metonymically indexing relevant emotion schemata, and 42 cases of emotion language (42,42%) explicitly mentioning an emotion. This indicates that, in the sample, emotions need to be more frequently imagined from the description of their physiological symptoms than explicitly constructed from an emotion term, even though this pattern is unevenly distributed across the two texts (Table 1).

**TABLE 1:** Narrated emotional states in “Harry” and *Atonement* chapter one.

	Harry	Atonement	Total
Emotion language	*N* = 27 (64,28%)	*N* = 15 (35,71%)	*N* = 42 (42,42%)
Physiological narrated perception	*N* = 44 (77,19%)	*N* = 13 (22,80%)	*N* = 57 (57,57%)
Total	*N* = 71 (71,71%)	*N* = 28 (28,28%)	*N* = 99 (100%)

As shown in Table 1, “Harry” contains a larger number of emotional state expressions characterizing Mrs. James (*N* = 71; 71,71%), compared to those characterizing young Briony in the novel chapter (*N* = 28; 28,28%), where the young girl’s emotions are not so frequently presented. Moreover, the frequency of occurrence of physiological narrated perception is overwhelmingly predominant in “Harry,” which contains 44 (77,19%) of the total 57 cases of emotional expressions found, compared to just 13 (22,80%) in the novel chapter. “Harry” also contains a larger number of lexical items explicitly labelling the felt emotion, but in a lower percentage: 27 (64,27%) versus 15 (35,71%) in the chapter. As a whole, it can be claimed that the focalizing character’s emotional state is more frequently narrated, either explicitly or through metonymic triggering, in the ghost story, where, additionally, this is predominantly achieved through physiological narrated perception.

### 6.1 Emotion language

Regarding the presence of language of emotion, that is, of lexical items explicitly naming the emotion felt by the character, the differences between the two texts are not only quantitative, but also qualitative. As shown in Table 2, the 27 cases of emotion language in “Harry” predominantly involve the basic emotion schemata FEAR (8 tokens) and ANGER (2) in the bereaved narrating mother, with a variety of secondary emotions (Anderson and Adolphs, 2014, 2018) such as HATE (3); LONGING (2); MADNESS (2); CONSOLATION (1); DESPAIR (1); FOOLISHNESS (1); LIGHT-HEARTEDNESS (1); LOSS (1); NERVOUSNESS (1); RELIEF (1); RESTLESSNESS (1); SURPRISE (1); and UNEASINESS (1). These emotion schema names are presented in capitals, to distinguish them from the lexical item(s) used in their textual realizations. In Table 2 these are presented by frequency, and, in cases of equal frequency, in order of appearance in the narrative.

**TABLE 2:** Emotion language in “Harry.”

Emotion	Token (27) (my italics)
FEAR (8)	Such ordinary things make me *afraid* (p. 33)
	I felt a premonition of *fear* (p. 33)
	I came to hate and *dread* those long summer days. (p. 36)
	Her blue eyes wide open and *frighteningly* cold (p. 40)
	The crazy eyes …*frightened* me. (p. 45)
	Mad people are *terrifying*. (p. 46)
	One can pity them, but one is still *afraid* (p. 46)
	I made *frantic* enquiries of passers-by (p. 46)
HATE (3)	I came to *hate* and dread those long summer days. (p. 36)
	*hating* the anger in my voice (p. 40)
	*hating* myself (p. 40)
LONGING (2)	I *longed* for grey skies and rain (p. 36)
	I *longed* for the white roses to wither and die (p. 36)
ANGER (2)	the *anger* in my voice (p. 40)
	hating myself for being *angry* (p. 40)
MADNESS (2)	Like a *mad* woman I opened the window and shouted (p. 40)
	*Demented*, I rushed out into the garden (p. 47)
UNEASINESS (1)	*Uneasy* without knowing why, I called her. (p. 33)
RELIEF (1)	It was a *relief* finally to tuck her up in bed (p. 34)
CONSOLATION (1)	I felt *consoled.* (p. 34)
DESPAIR (1)	I told myself *desperately* (p. 35)
SURPRISE (1)	The cry burst from me harshly, *surprising* me (p. 35)
NERVOUSNESS (1)	*it got more and more on my nerves* (p. 36)
FOOLISHNESS (1)	Jim made me feel *foolish* (p. 36)
RESTLESNESS (1)	I waited *restlessly*. (p. 38)
LOSS (1)	I felt a sense of *loss* at parting with her (p. 41)
LIGHT-HEARTEDNESS (1)	I felt quite *light-hearted* as I walked away (p. 42)

As can be observed in Table 2, 16 different emotion schemata seem to be explicitly prompted in “Harry” readers through the use of emotion language, and two of the most frequent – FEAR, ANGER – correspond to basic emotions. Since fear is one of the phylogenetic, basic human emotions (Gu et al., 2019; Zibin and Hamdan, 2019), the fact that it is explicitly triggered twice at the beginning of “Harry” is an effective strategy, unequivocally inviting readers to mentally construct and imagine a ghostly storyworld. Fear is once again strategically explicitly mentioned in the middle of the narrative (p. 40) and three times near the end (pp. 45−46). MADNESS seems to be presented as an emotional state in these particular occurrences, and has been classified accordingly (Theodorou, 2009). The same is the case with LOSS and FOOLISHNESS, which may refer to events or conditions in different contextual environments, but which, in this case, unequivocally index emotional states.

In *Atonement*’s chapter, however, there are only 15 cases of emotion language, and the emotion schemata involved are of a very different nature (Table 3). The most frequent are EMBARRASSMENT (2) and AMBITION (2), giving an idea of the completely different mental states of the character that focalizes the chapter, young Briony Tallis. These emotions are accompanied by SHAME (1), INFERIORITY (1), OVERWHELM (1), DISCOURAGEMENT (1), and SELF-PITY (1). Only two tokens relate to basic emotions, namely HORROR (1) for FEAR, and MISERY (1) for SADNESS. On a different note are LOVE (1) – for literature, not people, in the sample; YEARNING (1); COMPLIANCE (1); and RELIEF (1).

**TABLE 3:** Emotion language in *Atonement* chapter one.

Emotion	Token (15) (my italics)
EMBARRASSMENT (2)	Pretending in words was too tentative, too vulnerable, too *embarrassing.* (p. 6)
	Now her play seemed miserable, *embarrassing* (p. 13)
AMBITION (2)	It was not insensitivity so much as a highly focused artistic *ambition* (p. 9)
	They could never understand her *ambition* (p. 11)
YEARNING (1)	luminous, *yearning* fantasies. (p. 4)
RELIEF (1)	It was a *relief* not to be writing out … (p. 7)
LOVE (1)	This was precisely why she *loved* plays (p. 11)
SHAME (1)	Briony felt suddenly *ashamed* (p. 12)
INFERIORITY (1)	Briony felt *the disadvantage of being two years younger* than the other girl (p. 13)
OVERWHELM (1)	She proceeded to outline the plot, even as its stupidity began to *overwhelm* her (p. 13)
DISCOURAGEMENT (1)	She *no longer had the heart* to invent for her cousins the thrill… (p. 13)
HORROR (1)	unable to keep the *horror* from her expression (p. 13)
MISERY (1)	The *misery* of the inevitable was clouding her thoughts (p. 14)
COMPLIANCE (1)	and felt as she did so a sulky thrill of self-annihilating *compliance* (p. 14)
SELF-PITY (1)	*Self-pity* needed her full attention. (p. 15)

As Adolphs et al. (2019: 1) underscore, the scientific community still disagrees on how emotions “should be defined, on where to draw the boundaries for what counts as an emotion and what does not.” Most of the emotions identified above can be easily accepted as such. Others, however, such as AMBITION or INFERIORITY, seem to call for double-checking: ambition is considered one of the two central modern emotions, together with love (Greenfeld, 2013), while Liu et al. (2022: 1) describe the emotional nature of inferiority in these terms: “Feelings of inferiority are complex emotions that usually indicate perceived weakness and helplessness.” The explicit triggering of these emotion schemata in readers through the use of emotion language orients their mentalizing processes regarding character construction in the two data samples, and can be expected to contribute to the imaginative re-use of cause-effect relationships and expected behaviors remembered from previous real-world experiences, and re-combined and enriched to fit the simulation environment of the mentally constructed storyworld.

### 6.2 Physiological narrated perception and emotion schemata

The triggering of certain emotion schemata through the use of explicit lexical items described above makes it possible for readers to have immediate access to the most appropriate emotion and its related information as stored in their minds from previous experience. This does not guarantee that the said emotion schema has the same internal topology across readers, for, despite individuals’ reliance on socially-agreed emotional functions, the experiential nature of schemata involves a significant degree of individual variation (Rumelhart, 1980). Even so, the linguistic labelling of the character’s emotions afforded by the use of emotion language contributes to cognitive economy and facilitates processing. This is not the case, however, with the use of physiological narrated perception, which provides a description of the symptoms of the character’s emotional state, without explicitly specifying it. This inevitably imposes a stronger cognitive demand on readers, who have to not only retrieve relevant information from their own emotion schemata, but also decide which emotion is at stake, to begin with. And if schemata differ in their internal structure, setting up meaningful connections between certain physiological data and an associated emotional state is an even harder task. As Adolphs et al. (2019) explain:

“In a given moment, my brain might categorize my sense data as an instance of sadness and your brain might categorize the sense data coming from me — my movements, my vocal acoustics, in the same context — as an instance of anger. We can compare our inferences to one another, or what is normative in that particular situation, but there are no objective criteria to say who is right.” (Adolphs et al., 2019: 2).

In narrative experiences, readers tend to be familiar with the genre features of the text that they are reading, and this may help in this emotional state attribution process. In the two narratives in the present study, for instance, readers of “Harry” can be expected to know that this is a ghost story, since it is usually found in ghost story collections. This will probably contribute to their expecting fear to be a predictable emotion in the focalizing character(s), and in themselves. For this reason, it was hypothesized that “Harry” would contain a larger number of instances of physiological narrated perception than *Atonement* chapter one, as readers would find it easier to metonymically trace these symptoms back to the predictable emotion schemata associated with the ghost story genre. This hypothesis seems to be confirmed by the analysis, as shown in Table 1: 44 (77,19%) of the 57 cases of physiological narrated perception in the two data samples are found in “Harry,” while only 13 (22,80%) occur in the novel chapter. If what is considered is the relative presence of physiological narrated perception in each of the narrative samples, “Harry” ranks first again, since 44 (61,97%) of its 71 expressions representing emotional states are in this metonymic form, against 27 cases (38,02%) of explicit emotion language (Table 4). Conversely, the 28 expressions of the focalizing character’s emotional states in *Atonement* chapter one are more equally split into 13 cases of metonymic triggering (46,42%) and 15 cases of explicit emotion language (53,57%). This further confirms the research hypothesis, since it provides evidence that the presentation of the focalizing character’s emotional states in the novel resorts more consistently to the explicit labelling of the experienced emotion, seemingly to simplify readers’ efforts to activate appropriate emotions schemata, simultaneously minimizing the cognitively more complex task of metonymically inferring a relevant emotional state from the mere description of its physiological symptoms.

**TABLE 4:** Physiological narrated perception in “Harry” and *Atonement* chapter one.

	Harry	Atonement	Total
Emotion language	*N* = 27 (38,02%)	*N* = 15 (53,57%)	*N* = 42 (42,42%)
Physiological narrated perception	*N* = 44 (61,97%)	*N* = 13 (46,42%)	*N* = 57 (57,57%)
Total	*N* = 71 (100%)	*N* = 28 (100%)	*N* = 99 (100%)

As Anderson and Adolphs (2014: 188) observe, “Identifying instances of emotional expression is intuitively obvious to a lay person,” since these include, for example, “facial expressions such as frowning, vocalizations such as screaming or sobbing, and physiological expressions such as tearing or blushing.” However, as observed above, determining exactly which emotion schema is going to be triggered in each individual reader by the narrative presentation of these expressive behaviors in characters is quite an impossible enterprise. One can approximate the likelihood of a FEAR schema associated with the physiological process of “trembling” in “My hands were trembling” (Timperley, 1983[1955]: 45), derived from our bodily experiences of fear. But it is not impossible that some readers construct the emotion simply as intense worry, with tinges of distress, or anger, or impotence.

The difficulty in determining the exact nature of the emotion schemata to be triggered by these expressions in readers is better illustrated in Tables 5 and 6. Table 5 presents the instances of narrated physiological perception in “Harry.” As can be observed, the 44 narrated physiological processes in this ghost story involve the activity of a variety of invisible systems, more specifically the vocal (10), motor (8), muscular (6), homeostatic for body temperature (6), nervous visual (3), respiratory (1), and cardio-vascular (1). Additionally, there are emotional behaviors which involve certain paralinguistic features of speech (9), such as stammering, sudden interruptions, or shouting and screaming, which are also customarily associated with emotional states. However, as pointed out above, the specific emotion schemata metonymically triggered by the narrated presentation of these emotional behaviors is difficult to establish. As can be observed in Table 6, the presence of physiological narrated perception in *Atonement* chapter one is significantly lower, with just 13 cases. The most frequent here are of a motor type (4), followed by those involving the cardio-vascular (2), muscular (2), nervous visual (2), vocal (2), and respiratory (1) systems. These narrated descriptions of emotion-related physiological symptoms, however, do not always unequivocally evoke a given emotion. Rather, this metonymic pointing is strongly dependent on the surrounding circumstances and context, both fictional and real, since features of both the fictional context of the storyworld, and the reader’s genre expectations and idiosyncratic personal experience and circumstances (Martínez, 2018) will have a bearing on which emotion schema(ta) are metonymically activated.

**TABLE 5:** Physiological narrated perception in “Harry.”

Narrated expressive behavior	Token (44)
Vocal (10)	*The cry burst from me harshly.* (p. 35)
	“…” *I blurted out* (p. 36)
	*I couldn’t say the name.* (p. 36)
	“…” *I laughed ruefully* (p. 36)
	“Don’t!” *I cried*. “Don’t say that!” (p. 36)
	“…” I said *quietly* (p. 38)
	“…,” *I shouted* (p. 40)
	I opened the window and *shouted* “Harry! Harry!” (p. 40)
	“…,” *I murmured* (p. 44)
	my own voice of the past *screaming.* (p. 47)
Paralinguistic (stammering, screaming) (9)	“Who can she be getting it from except *Ha*…” (p. 36)
	“It’s only when she’s talking *to – to* him.” (p. 36)
	“*I’m – I’m* afraid of it, Jim.” (p. 37)
	*“Chris, stop this nonsense! Stop it!”* (p. 40)
	“…a little girl – *my* little girl (italics in the original)” (p. 40)
	“What *did – her brother – say*?” (p. 46)
	“*Chris! Christine, where are you? Chris! Chri*s!” (p. 47)
	“…*Chrrriiiiiss!*” (p. 47)
	“*Come back! Harry! Harry!*” (p. 47)
Motor (8)	I … *held her hand tightly all the way home* (p. 39)
	*I never let her out of my sight.* (p. 39)
	*I struck her sharply on the arm* (p. 40)
	*I went down on one knee and held out my arms* (p. 40)
	But one thing made me *stare and stare.* (p. 44)
	*I stumbled on* (p. 46)
	*I ran all the way home* (p. 46)
	*I rushed out into the garden* (p. 47)
Muscular (6)	*My hands were trembling* (p. 35)
	*I trembled* when I heard Christine’s voice… (p. 36)
	“…,” I said *shakily* (p. 38)
	She gave me a stare that *made me tremble* (p. 40)
	*my legs felt heavy and half-paralyzed* (p. 45)
	“.” I asked *faintly.* (p. 46)
Homeostatic (body temperature) (6)	*I felt myself going cold* as I stood there in the kitchen (p. 35)
	*It was chilly* in the house nowadays (p. 35)
	the house *so strangely cold* in this hot weather… (p. 39)
	through *the cold house* (p. 46)
	through *the burning streets*. (p. 47)
	*The sun struck me like a hot blade. (p. 47)*
Lacrimal and visual nervous (3)	*I was almost in tears* (p. 41)
	*The world turned red. Blood red. Wet red.* (p. 47)
	I felt *through redness and blackness to nothingness* (p. 47)
Respiratory (1)	“…” *I gasped* (p. 46)
Cardio-vascular (1)	Then I heard something that *chilled my blood* (p. 46)

**TABLE 6:** Physiological narrated perception in *Atonement* chapter one.

Narrated expressive behavior	Token (13)
Motor (4)	*unable to keep the horror from her expression* (p. 13)
	*unable to speak* (p. 13)
	*unable to push her tongue against the word* (p. 14)
	Briony *could only nod* (p. 14)
Cardio-vascular (2)	she made *her heart thud* with luminous, yearning fantasies (p. 4)
	…*her heart thudded* inconveniently (p. 15)
Muscular (2)	made her *wince* (p. 6)
	The sight of these nails gave Briony *a constricting sensation around her sternum* (p. 11)
Visual nervous (2)	and out into *the blinding light of midday* (p. 9)
	*darkening the room in throbs* (p. 14)
Vocal (2)	and said in *a voice that was constricted and more high- pitched than usual*, “…” (p. 15)
Respiratory (1)	“If you are Arabella, then I’ll be the director, *thank you very much*,…” (p. 15)
	*her breath was short* (p. 15)

Consider, for instance, the case of the emotion schema for FEAR. As observed above, in “Harry” this emotion is the most frequently labelled through explicit emotion language. Fear is one of the basic emotions, so that the vast majority of the physiological expressions in Table 5 – stammering and screaming, trembling, half-paralyzed limbs, a chilly sensation – can be readily identified as bodily symptoms associated with this emotion, particularly in the telling of a ghost story. However, the emotions explicitly labelled through the use of emotion language in *Atonement* chapter one are more complex and often hybrid, secondary emotions. Accordingly, the emotional behaviors presented in Table 6 cannot easily be pinned down as metonymically pointing to a specific emotional state, not even when found in their surrounding co-text. Consider, for instance, the physiological perception narrated in “The sight of these nails gave Briony *a constricting sensation around her sternum*” (McEwan, 2002, p. 11) (my italics). It must be remembered that the narrative beginning in McEwan’s novel prompts the activation of readers’ past ‘pre-adolescent’ selves (Martínez, 2018) when deictically shifting into the storyworld to share vantage point with young Briony. The memories structuring this mental representation of a past self will vary from reader to reader, so that the exact emotions associated with seeing a two-year-older girl look so womanly when you yearn for others to see you no longer as a child, are also going to be different. Furthermore, “a constricting sensation around (the) sternum” is a physiological symptom that might as well be felt in a number of other situations, such as before an important job interview, to mention a previous example. Adding to this, this probably familiar sensation is also ambiguous in its metonymic association with a given emotion schema even in a single context, not just because of linguistic ambiguity, but mostly because, as human beings, we ourselves often find it difficult to exactly identify the emotion that we may be feeling when we bodily experience a somatomotor constriction of the sternum. This ambiguity results in increased opportunities for readers’ idiosyncratic, personally relevant meaning construction, grounded on the emotional mental imagery with which such a sensation is associated, and the emotion schemata in which it is present. Moreover, the exact internal topology of these emotion schemata will vary from reader to reader too, as happens with mental representations at large (Bartlett, 1932; Rumelhart, 1980).

Something similar happens with the two cases of vocal physiological perception in *Atonement* chapter one (Table 6), narrated in the following extract from the novel:

(9) She (Briony) took the play from Lola and said in a voice that was *constricted* and more *high-pitched* than usual, “If you’re Arabella, then I’ll be the director, thank you very much, and I’ll read the prologue”. (McEwan, 2002, p. 15). (my italics).

Here, the character’s *constricted* and *high-pitched* voice gains its significance in the context of the episode in which it occurs: young Briony has made all sorts of arrangements to play the leading role of princess Arabella in her play, to impress her family, but her older cousin Lola has manipulated the situation to play that role herself, something which Briony, an only child with little experience of being cast in a secondary position, finds completely annoying and difficult to respond to. A breaking of Grice’s Quality maxim in that high-pitched “thank you very much” – there is nothing to be thankful for, rather the opposite – and the expressive implicature which it involves, reinforce the physiological vocal cues with which the narrator invites readers to construe the character’s emotions at this point. But, again, the exact emotion schemata activated in individual readers are likely to differ in many respects, due to the idiosyncratic nature of schema construction, and to the multivalent nature of many physiological emotional manifestations (Stemmler, 2004; Adolphs et al., 2019). Moreover, complexity in this case is intensified by the ambiguous perceptual viewpoint resulting from the absence of a verb of perception: is this Briony’s perception, since she is the internal focalizer in this extract, or is it the omniscient narrator’s? While in “Harry” the use of a first person narrator made it clear at all points that these physiological perceptions are the character’s, in *Atonement* chapter one readers are forced to further infer whether this is the character’s physiological perception, or the narrator’s auditory one. The emotional complexity of McEwan’s novel and its stronger cognitive demands thus become even more evident if physiological narrated perception is incorporated in the analysis of its perceptual viewpoint(s).

One final observation derived from this research concerns the frequent co-occurrence of physiological narrated perception with cases of narrated perception (NP) of a sensorial nature, namely visual, auditory, olfactory, tactile, or gustatory, as in example (10):

(10) There were moments *in the summer dusk after her light was out* (visual NP), *burrowing in the delicious gloom of her canopy bed* (tactile, visual NP), when *she made her heart thud* (physiological NP) with luminous, yearning fantasies. (McEwan, 2002, p. 4) (my italics).

As can be observed, the visual narrated perception of the gloom in Briony’ room before sleep, and the tactile perception of the enveloping warmth of the bedclothes around her, accompanies the physiological narrated perception of how her heart thuds, and the explicit use of emotion language to label the YEARNING emotion schema associated with the girl’s dreamy fantasies. This seems to provide further evidence for the multimodal nature of sensorial imagery (Starr, 2010; Lacey and Lawson, 2013; Otis, 2022). The tight connections between the narrated presentation of a focalizer’s physiological processes and of other types of sensorial perception suggest that all of them jointly contribute to linguistically encouraging readers’ embodied experience of the storyworld through the activation of contextually relevant emotion schemata and associated emotional mental imagery.

To sum up, the analysis of the presence of emotion language and physiological narrated perception in the two data samples, and of its connection to the activation of emotion schemata in readers, shows a stronger reliance on the implicit, metonymic triggering of emotion schemata in Timperley’s ghost story “Harry,” compared to the larger presence of explicit emotion language in *Atonement* chapter one, thus confirming the study’s research hypothesis. Since physiological triggering does not unambiguously specify the emotion schema to be activated by readers, these are likely to have to rely more overtly on embodied personal experience and genre familiarity in the ghost story, particularly in connection with their emotion schema FEAR. In McEwan’s novel, on the contrary, a higher reliance on the use of emotion language to explicitly label the emotional states of the focalizing character seems to more overtly orient the projection of relevant emotion schemata in readers, in order to facilitate their navigation of the novel’s emotional complexity. In the few cases in which this is not the case, that is, in which the focalizing character’s emotions have to be inferred on the basis of her emotional behavior, emotional ambiguity itself seems to become a part of the very fabric of the author’s narrative technique, so that readers can generate personally relevant emotional mental imagery. This remembered mental imagery can be imaginatively re-used in novel, self-relevant ways, within the fictional circumstances which surround the storyworld deictic center shared with the narrative perspectivizer. In this way, emotions similar to those associated with a pre-experienced physiological symptom can now be attributed to the focalizer through Theory of Mind associations, and re-combined with features of the simulation environment of the mentally constructed storyworld. But identification with the focalizer is not a necessary condition, because the metonymic physiological trigger may, simply, evoke a personally felt emotion which is not shared with the narrative entity, but which may generate self-relevant emotional mental imagery, with increased opportunities for feelings of engagement.

## 7 Conclusion

This study has explored the narrative prompting for mental imagery through the presentation of narrative focalizers’ perceptions of their own physiological processes as symptoms of emotional states, and as metonymic triggers for readers’ mental construction of those states on the basis of the personal experience stored in the mind in the form of emotion schemata. These emotion schemata contain all the background information about an emotion that the individual has acquired through personal and socio-cultural experience, and include the mental imagery stored in memory together with the events and situations in which the emotion was felt.

During a narrative experience, these imagined emotions can be re-used and re-combined in contextually relevant ways which, in the analysis, seem to be significantly influenced by genre expectations. The study has focused on the identification of what I have called *physiological narrated perception* in two literary samples of similar lengths – the ghost story “Harry” (Timperley, 1983[1955]) and the first chapter in Ian McEwan’s novel *Atonement* (2002). The results highlight the narrative role of these expressions which, by describing the activity of the invisible systems – muscular, motor, respiratory, cardiovascular, homoestatic – seem to invite readers to imagine and share the characters’ emotional states when faced with certain emotion-provoking events and situations in the storyworld. This suggests that physiological narrated perception can be considered a further case of narrated perception in its own right, to be added to the five acknowledged types – visual, auditive, olfactory, tactile, gustatory - which traditionally account for the description of perceptions of an external sensorial nature. This inclusion is reinforced by textual evidence of the frequent co-occurrence of physiological narrated perception with other types of sensorial description, which indicates that the embodied experience of storyworlds prompted by fictional narratives is of a holistic nature, and includes both external and internal perception.

Moreover, given the difficulty in associating a certain physiological response to a specific emotion, a phenomenon consistently reported in psychology and neuroscience, physiological narrated perception shares with other narrated perception types the potential to generate mental imagery of an emotional nature, probably based on remembered experience, but revisited anew to fit the simulation environment circumstances of a mentally constructed storyworld. Revisiting an emotion from the perspectival viewpoint of a fictional other is an embodied experience which must strongly call upon the human capacity to imagine emotions on the basis of textual clues, among which the narrated description of physiological perception, or the perception of the workings of the body’s invisible systems, is of paramount importance, and may provide a better understanding of the cross-fertilization between emotional mental imagery and narrative experiencing. The key role played by physiological narrated perception in this mutual feeding is probably due to its intrinsic ambiguity and multi-indexicality, and to the multiplicity of emotional mental images that is has the power to evoke, so that its study can further illuminate the ways in which narrative experiencing and the human capacity to imagine may feed on each other.

## Data availability statement

The original contributions presented in the study are included in the article/supplementary material, further inquiries can be directed to the corresponding author.

## Author contributions

M-AM: Funding acquisition, Writing – original draft.
